# Polymorphisms in *CTLA4* Influence Incidence of Drug-Induced Liver Injury after Renal Transplantation in Chinese Recipients

**DOI:** 10.1371/journal.pone.0051723

**Published:** 2012-12-21

**Authors:** Yifeng Guo, Yu Fan, Jianxin Qiu, Yong Liu, Junwei Gao, Fang Guo

**Affiliations:** 1 Organ Transplantation Center, Shanghai First People's Hospital, School of Medicine, Shanghai Jiao Tong University, Shanghai, China; 2 Department of Pharmacy, Shanghai First People's Hospital, School of Medicine, Shanghai Jiao Tong University, Shanghai, China; 3 Center for Nanomedicine and Translational Medicine, Shanghai Advanced Research Institute, Chinese Academy of Sciences, Shanghai, China; Oslo University Hospital, Norway

## Abstract

Genetic polymorphisms in cytotoxic T lymphocyte-associated antigen 4 (CTLA4) play an influential role in graft rejection and the long-term clinical outcome of organ transplantation. We investigated the association of 5 CTLA4 single-nucleotide polymorphisms (SNPs) (rs733618 C/T, rs4553808 A/G, rs5742909 C/T, rs231775 A/G, and rs3087243 G/A) with drug-induced liver injury (DILI) in Chinese renal transplantation (RT) recipients. Each recipient underwent a 24-month follow-up observation for drug-induced liver damage. The CTLA4 SNPs were genotyped in 864 renal transplantation recipients. A significant association was found between the rs231775 genotype and an early onset of DILI in the recipients. Multivariate analyses revealed that a risk factor, recipient rs231775 genotype (p = 0.040), was associated with DILI. Five haplotypes were estimated for 4 SNPs (excluding rs733618); the frequency of haplotype ACGG was significantly higher in the DILI group (68.9%) than in the non-DILI group (61.1%) (p = 0.041). In conclusion, CTLA4 haplotype ACGG was partially associated with the development of DILI in Chinese kidney transplant recipients. The rs231775 GG genotype may be a risk factor for immunosuppressive drug-induced liver damage.

## Introduction

In renal transplantation, immunosuppressive therapy is usually administered as a triple regimen, such as cyclosporine A (CsA)/tacrolimus (TAC)+mycophenolate mofetil (MMF)+prednisone (Pred). The triple regimen is favored because it produces a more effective immunosuppression and lessens the drug-induced damages or side effects. However, complications such as leukopenia, drug-induced liver injury (DILI), osteoporosis, infection and tumors often appear [1]–[5].

Cytotoxic T lymphocyte-associated antigen 4 (*CTLA4*) is a key element in the immune system that induces immune tolerance and is one of the critical negative regulators of the T cell-mediated immune response [6]. It is also expressed constitutively on the surface of regulatory T cells (Tregs) and is detectable on approximately 50% of Tregs; it is only found on <1% of naive helper T cells [7]. *CTLA4* ligation on Tregs results in a significant decrease in the presentation capacity of antigen-presenting cells and effector T cell downregulation in mice [8]. *CTLA4* plays an important role in the downregulation of the immune response. The rs231775 (+49A/G) SNP is located within the signal peptide of the molecule and influences expression of the full length isoform on the T cell membrane. The expression pattern of the CTLA-4 protein was found to be changed by a polymorphism of the rs4553808 (−1661A/G) and rs5742909 (−318C/T) genotypes, located in the CTLA-4 gene promoter [9]. Similarly, the rs733618 (−1772T) allele was found to decrease the transcription level of the CTLA-4 gene by influencing the binding of transcription factors [10]. The rs3087243 (+6230G/A) SNP is situated within the 3′ untranslated region of the CTLA-4 gene and was found to be associated with susceptibility to autoimmune diseases [11]. The single-nucleotide polymorphisms (SNPs) of the *CTLA4* gene +49A/G (rs231775) and +6230 G/A (rs3087243) play an influential role in graft rejection and the long-term clinical outcome of organ transplantation [12]–[16].

*CTLA4* gene polymorphism has been suggested to influence liver damage. Kanno et al [17] discovered that SNP *CTLA4* +49GG (rs231775) may be associated with the liver damage of primary biliary cirrhosis (PBC) in Japanese populations. Valenti et al [18] observed a significantly higher prevalence of subjects carrying the *CTLA4* susceptibility allele (both in the heterozygous and homozygous states) among patients with ALD compared to healthy subjects. The *CTLA4* polymorphic G allele may confer susceptibility to ALD and, in the homozygous state, to alcoholic cirrhosis.

The role of *CTLA4* SNPs in T cell mediated immunity post-transplantation and in the condition of drug-induced liver injury is unknown. Therefore, this study was designed to investigate the associations between five *CTLA4* SNPs (rs733618 C/T, rs4553808 A/G, rs5742909 C/T, rs231775 A/G, and rs3087243 G/A) and drug-induced liver injury (DILI) in Chinese renal transplantation recipients.

## Materials and Methods

### Diagnostic criteria and methods

A grade ≥14 on the scale reported by Maria et al [19] was used to diagnose DILI. DILI was suspected in patients with symptomatic liver disease and those with asymptomatic elevations in liver function tests (LFTs). LFT abnormalities were categorized into hepatitic, cholestatic or mixed based on abnormalities of serum alanine aminotransferase (ALT) and serum alkaline phosphatase (ALP) and the relationships of these to their respective upper limits of normal (ULNs). Liver damage was categorized according to the US Food and Drug Administration hepatotoxicity steering committee [20] : hepatitic pattern = ALT>3 ULN & [(ALT/ULN)/(ALP/ULN)]>5; cholestatic pattern = ALP>2 ULN & [(ALT/ULN)/(ALP/ULN)]<2; mixed pattern = ALT>3 ULN & ALP<2 ULN and [(ALT/ULN)/(ALP/ULN)]>2 but <5.

People having the following conditions were excluded from the study: hepatitis caused by excessive consumption of alcohol; presence of hepatitis virus A, B, C, D or E; fatty liver; autoimmune hepatitis; hereditary liver disease; hemorrhagic or congestive hepatitis; hyperthyroidism liver injury; non-hepatotropic viral hepatitis; or hepatitis of another cause.

### Patients

This study included 864 transplantation recipients (764 cadaver donor cases and 100 living donor cases; 536 men and 328 women) in the Shanghai Organ Transplantation Center between Jan 2000 and Oct 2011. Ninety patients had DILI, and 774 cases had no liver injury. Of the 90 patients with DILI, 32 cases presented with a primarily hepatitic pattern, 36 with a cholestatic pattern and 22 with a mixed pattern. The mean age of the patients included in the study was 40.09±10.06 years. Overall, 816 cases of chronic glomerulonephritis, 25 cases of polycystic kidney disease, and 23 cases of pyelonephritis were detected. Preoperative negativity for all hepatitis viruses and a normal liver function were required. All of the recipients were blood group-matched with their donors and were tested for the panel-reactive antibody and HLA-A-B-DR matching.

Each organ donation or transplant in our center was strictly selected according to the guidelines of the Ethical Committee review board of our hospital, the regulation of Organ Transplant Committee of Shanghai Jiao Tong University and the Declaration of Helsinki. The study protocol was approved by the Ethical Committee review board of our hospital and Shanghai Jiao Tong University and informed. The research process was explained to every candidate patient from the collection and storage of blood, isolation of DNA and determination of gene polymorphisms in detail. Every participant gave written informed consent form. The Ethical Committee review board of our hospital and Shanghai Jiao Tong University approved this consent procedure and the study.

This is a cross-sectional study. Each patient underwent a 24-month follow-up observation through which clinical information was provided by means of clinical observation, medical records and outpatient or telephone follow-up visits. The exclusion criteria were (1) observed time less than 24 months, (2) die with other reasons (infection, etc) in 24 months post-transplantation, (3) stop using immune suppressants with graft function failure, and (4) not able to provide written informed consent. This study was performed from Jan 2000 to Oct 2011; all patients discharged in about 30days after operation and visited our outpatient clinics; all patients that did not meet the exclusion criteria (n = 864) were invited to take part in the present study.

### Immunosuppression protocol

Mycophenolate mofetil (MMF) 1.0 was given as a premedicant. Intravenous infusion of 500 mg/d of methylprednisolone was applied during the procedure through 2 days after the operation. The dose was then decreased to 360 mg, 180 mg, 80 mg and 40 mg each subsequent day, followed by prednisone (15–20 mg/d) as a maintenance therapy. Triple therapy with cyclosporine A (CsA)/tacrolimus (TAC), MMF and prednisone was administered beginning on the third day after the operation. The dosage of MMF was 1.0–1.5 g/d with a weight of 60 kg as the critical value. CsA and TAC were started at doses of 8 mg/kg/d and 0.2 mg/kg/d, respectively, and then adjusted according to the plasma concentrations and the serum creatinine concentrations.

The diagnostic criteria of AR were based on the comprehensive elevation of histological and clinical symptoms, their alleviation by anti-rejection therapy and graft biopsy. The clinical symptoms examined were hypourocrinia, fever, weight gain, pain in the transplanted kidney, elevated blood pressure, increased serum creatinine (to 25% above baseline), urine protein and the resistance index. The Banff 97 working classification for renal allograft pathology (modified) [21] was used as the pathological rejection criteria.

### Sample collection and polymorphism genotyping

A total of 864 patients were included in this study. Peripheral blood samples (3 ml) were collected, the DNA was extracted, and the SNPs of *CTLA4* were genotyped using polymerase chain reaction (PCR) and direct sequencing. The primers and annealing temperatures (ATs) employed for rs733618 C/T, rs4553808 A/G, rs5742909 C/T, rs231775 A/G and rs3087243 G/A were displayed in Table 1.

**Table 1:** PCR primers of the *CTLA4* SNP used in the study.

Locus	AT (°C)	Primer pairs (5′→3′)	Amplicon size (bp)
rs733618, rs4553808	58	CTAAGAGCATCCGCTTGCACCT	486
		TTGGTGTGATGCACAGAAGCCTTTT	
rs5742909	56	AAATGAATTGGACTGGATGGT	226
		TTACGAGAAAGGAAGCCGTG	
rs231775	58	GCTCTACTTCCTGAAGACCT	162
		AGTCTCACTCACCTTTGCAG	
rs3087243	59	AGGAAGGCAGATCAAAATGC	202
		CACCACTATTTGGGATATAACA	

AT: annealing temperature.

### Statistical analysis

Comparisons of clinical characteristics between patients with DILI and non-DILI were analyzed by the Pearson *χ^2^* test and an independent-sample test. We assessed the Hardy–Weinberg equilibrium (HWE) for both DILI and non-DILI using the *χ^2^* test. For linkage disequilibrium (LD), Haploview version 4.2 software was used [22]. A correlation test was used to assay the degree of correlation between DILI and AR. Genotype associations were analyzed using a dominant model (minor-allele homozygotes plus heterozygotes vs. major-allele homozygotes), a recessive model (minor-allele homozygotes vs. heterozygotes plus major-allele homozygotes) and a codominant model (minor-allele homozygotes and heterozygotes vs. major-allele homozygotes). The allelic frequencies were counted in a single strand of measured DNA. The differences in the genotype distributions between groups were analyzed by the *χ^2^* test or Fisher's exact test. According to presence and absence of acute rejection (AR), subanalysis was used by Chi-square test. The time of the first abnormal laboratory result indicative of DILI was designed as the post-transplantation time (days) of first abnormalities in liver function tests (LFTs) in recipients suffering from DILI and as an early onset of DILI. Associations of the *CTLA4* SNPs with an early onset of DILI in patients were analyzed by the Kaplan-Meier test. Multivariate analyses, logistic regression, were used to analyze several risk factors, including age, gender, primary diseases, number of HLA mismatches, acute rejection, blood transfusion, *CTLA4* SNPs. These risk factors were analyzed together. We explored the haplotype association for 5 SNPs using Haploview version 4.2. All statistical tests were two-sided, and statistical significance was set at *p*<0.05. Correction for multiple testing was carried out using the Bonferroni method. Statistical analysis was performed with SPSS (Statistical Package for the Social Sciences) version 11.5 software (SPSS Inc., Chicago IL, USA). All statistical tests were two-sided, and statistical significance was set at *p*<0.05.

## Results

### Baseline characteristics of 864 renal transplant recipients

The total number of patients was 864, with 536 male and 328 female cases. A total of 10.42% recipients (90/864) had DILI during the first 24 months post-transplantation. Baseline characteristics of 864 renal transplant recipients and types of DILI were listed in Table 2. No significant differences in age, sex, primary diseases, human leukocyte antigen mismatches, blood transfusion, renal transplantation or immunosuppressant regimen were found between patients with DILI and those without (Table 2). The incidence of acute rejection (AR) following renal transplantation was not different between the two groups (*p* = 0.053).

**Table 2:** Comparison of clinical characteristics between patients with DILI and non-DILI.

Characteristic	Patients with DILI (n = 90) (%)	Patients with non-DILI (n = 774) (%)	*p* value
Mean age±SD	40.967±10.361	39.986±10.008	0.602
Sex			
Male	56(62.22)	480(62.016)	
Female	34(37.78)	294(37.984)	0.969
Primary diseases			
Chronic glomerulonephritis	87(96.67)	729(94.19)	
Polycystic kidney	2(2.22)	23(2.97)	
Pyelonephophritis	1(1.11)	22(2.84)	0.557
Number of HLA -mismatch	2.51±0.927	2.48±0.829	0.156
Immunosuppressant regimens			
CsA+MMF+Pred	55(61.11)	513(66.28)	
TAC+MMF+Pred	35(38.89)	261(33.72)	0.799
Blood transfusion	11	93	0.955
Rejection			
AR/non-AR	20/70	112/662	0.053
Type of DILI			
Hepatitic pattern	32(35.6)	-	-
Cholestatic pattern	36(40.0)	-	-
Mixed pattern	22(24.4)	-	-

CsA: cyclosporine, MMF: mycophenolate mofetil, Pred: prednisone, TAC: tacolimous, AR: acute rejection, non-AR: non-acute rejection, DILI: drug induced liver injury.

**Figure 1:**
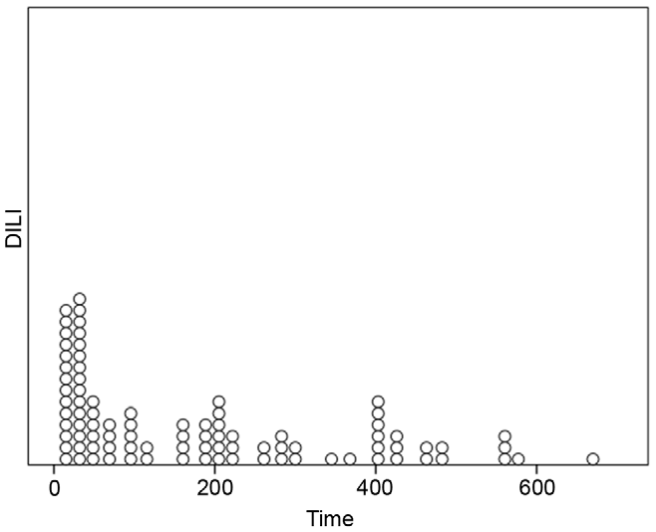
Diagnosis time of drug-induced liver injury after renal transplantation. Twenty-three patients were diagnosed drug-induced liver injury within the first month after operation; 26, 22 and 19 cases presented with DILI between 2 and 6 months, 7 and 12 months and 13 and 24 months after operation, respectively.

Twenty-three patients were diagnosed as having drug-induced liver injury within the first month after operation; 26, 22 and 19 cases presented with DILI between 2 and 6 months, 7 and 12 months and 13 and 24 months after operation, respectively. The scatter plot in Figure 1 showed the distribution of the patients with DILI throughout the entire observation period.

### Associations between the *CTLA4* SNPS and DILI

All polymorphisms were in Hardy-Weinberg equilibrium. Using Haploview version 4.2 software, the five loci were found to be in linkage disequilibrium (LD) (D' = 0.900–1.000). Regarding the genotype distribution of the *CTLA4* polymorphisms, no statistical differences for rs733618, rs4553808, rs5742909 or rs3087243 were found between patients with DILI and those without. However, the frequency of the rs231775 GG genotype in recipients with DILI was significantly higher (48.89%) than in those recipients without DILI (37.73%) (*p* = 0.040, OR = 1.579, 95% CI = 1.019–2.447, Bonferroni-adjusted *p* = 0.20) (Table 3).

**Table 3:** The genotype distribution of the *CTLA4* polymorphisms in patients with DILI and non-DILI.

Locus	Genotype	Patients with DILI (n = 90) n(%)	Patients with non-DILI (n = 774) n(%)	Model	OR (95% CI)	*p* value
rs733618	TT	42(46.67)	282(36.44)	Dominant	1.527(0.984–2.369)	0.058
	CT	36(40.00)	384(49.61)	Recessive	0.949(0.500–1.801)	0.872
	CC	12(13.33)	108(13.95)	Codominant	0.746(0.378–1.471)	0.396
					0.629(0.393–1.008)	0.053
rs4553808	AA	63 (70.00)	501(64.73)	Dominant	1.271(0.791–2.043)	0.320
	AG	21(23.33)	231(29.84)	Recessive	1.245(0.514–3.015)	0.627
	GG	6(6.67)	42(5.43)	Codominant	0.859(0.351–2.105)	0.740
					0.720(0.429–1.208)	0.212
rs5742909	CC	64(71.11)	512(66.15)	Dominant	1.260(0.780–2.035)	0.345
	CT	23(25.56)	241(31.14)	Recessive	1.236(0.361–4.230)	0.735
	TT	3(3.33)	21(2.71)	Codominant	0.875(0.254–3.016)	0.743
					0.763(0.463–1.259)	0.289
rs231775	GG	44(48.89)	292(37.73)	Dominant	1.579(1.019–2.447)	0.040
	AG	37(41.11)	383(49.48)	Recessive	0.758(0.369–1.557)	0.449
	AA	9(10.00)	99(12.79)	Codominant	1.658(0.781–3.517)	0.184
					0.641(0.404–1.019)	0.058
rs3087243	GG	73(81.11)	599(77.39)	Dominant	1.255(0.721–2.183)	0.422
	AG	14(15.56)	154(19.90)	Recessive	1.236(0.361–4.230)	0.735
	AA	3(3.33)	21(2.71)	Codominant	1.280(0.294–5.569)	1.000
					0.799(0.446–1.432)	0.451

DILI: drug induced liver injury, OR: odds ratio, CI: confidence intervals.

No differences in the determined allelic frequencies of rs733618, rs4553808, rs5742909 or rs3087243 were found between DILI and non-DILI recipients (Table 4). The allelic distribution of the locus rs231775 was not different between recipients with DILI and those without DILI (*p* = 0.066, OR = 1.366, 95% CI = 0.978–1.906).

**Table 4:** The allele distribution of *CTLA4* polymorphisms in patients with DILI and non-DILI.

Locus	Allele	Patients with DILI (n = 180) n(%)	patients with non-DILI (n = 1548) n(%)	OR (95% CI)	*p* value
rs733618	T	120(66.67)	948(61.24)	0.790(0.570–1.095)	0.156
	C	60(33.33)	600 (38.76)		
rs4553808	A	147(81.67)	1233(79.65)	0.879 (0.591–1.307)	0.523
	G	33(18.33)	315(20.35)		
rs5742909	C	151(83.89)	1265(81.72)	1.165(0.767–1.769)	0.474
	T	29(16.11)	283(18.28)		
rs231775	G	125(69.44)	967(62.47)	1.366(0.978–1.906)	0.066
	A	55(30.56)	581(37.53)		
rs3087243	G	160(88.89)	1352(87.34)	1.160 (0.712–1.890)	0.552
	A	20(11.11)	196(123.66)		

DILI: drug induced liver injury, OR: odds ratio, CI: confidence intervals.

In subanalysis, in which correction for multiple testing was carried out using the Bonferroni method, no statistical differences in genotype distribution and allelic frequencies of the *CTLA4* polymorphisms were found yet in AR group and non-AR group (Table S1, S2, S3 and S4).

**Figure 2:**
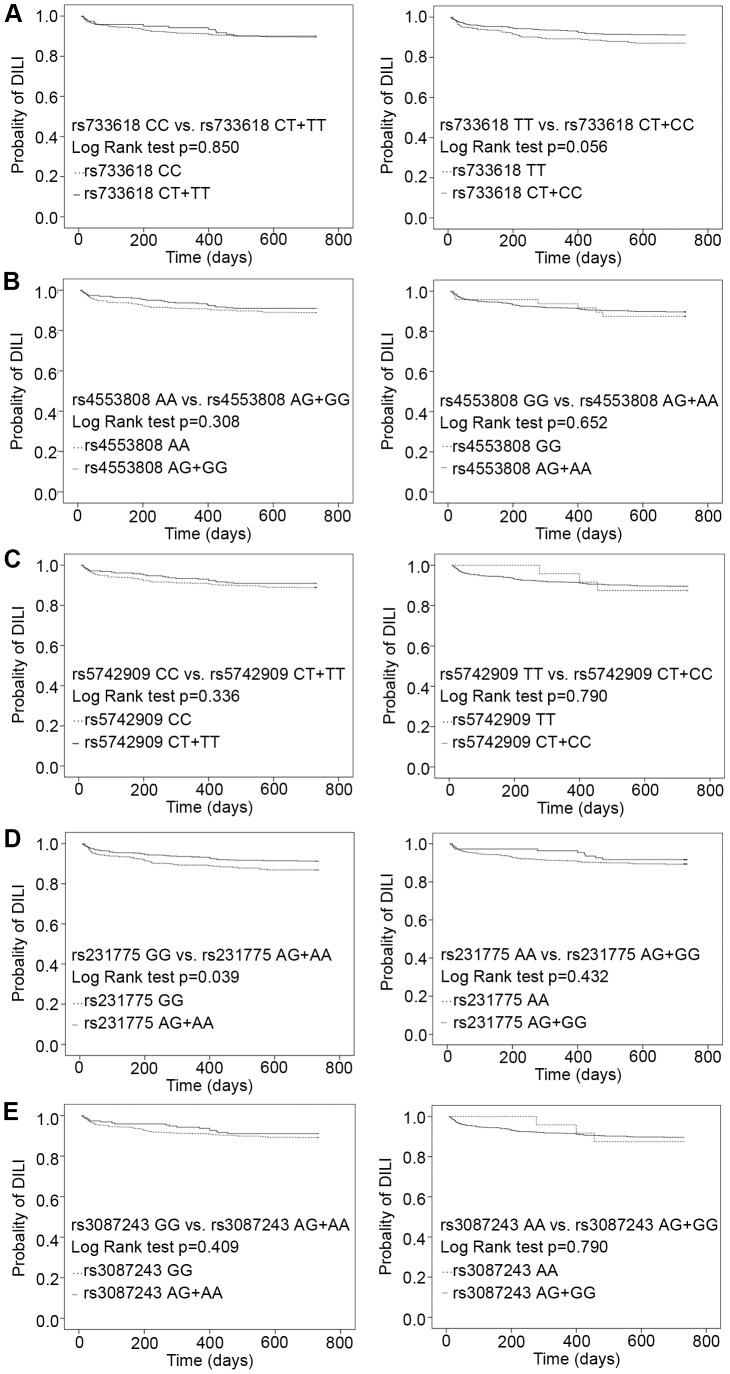
Association between *CTLA4* SNPs and early onset of drug-induced liver injury in renal transplantation. No statistical differences for rs733618 (Figure 2a), rs4553808 (Figure 2b), rs5742909 (Figure 2c) or rs3087243(Figure 2e) was found between DILI and non-DILI recipients. A significant difference (p = 0.039) was found between patients bearing the rs231775 GG genotype and those with the AA+AG genotypes using the log-rank test (Figure 2d).

Kaplan-Meier analysis was used to examine the relationships between *CTLA4* SNPs and an early onset of DILI (Table 5); no statistical differences for rs733618, rs4553808, rs5742909 or rs3087243 existed between DILI and non-DILI recipients. A significant difference (*p* = 0.039) was found between patients bearing the rs231775 GG genotype and those with the AA+AG genotypes using the log-rank test (Figure 2). Values of mean and 95% CI for the GG and AG+AA groups were 657.438±10.755 (95%CI: 636.358–678.517) days and 682.803±7.030 (95%CI: 669.024–696.592) days respectively. A significant association was found between the rs231775 genotype and an early onset of DILI in recipients.

**Table 5:** Correlation between early onset of DILI and *CTLA4* genotypes in recipients.

Locus	Genotype	Patients with DILI (n = 90) n(%)	Patients with non-DILI (n = 774) n(%)	Total counts (n = 864)	Means (days)	95%CI	*p* value*
rs733618	TT	42(46.67)	282(36.44)	324	658.022±10.918	636.622–679.421	0.056
	CT+CC	48(53.33)	492(63.56)	540	681.889±7.013	668.144–695.634	
	CC	12(13.33)	108(13.95)	120	680.400±14.671	651.644–709.156	0.850
	CT+TT	78(86.67)	666(86.04)	744	671.735±6.565	658.869–684.602	
rs4553808	AA	63 (70.00)	501(64.73)	564	667.054±7.836	652.144–682.863	0.308
	AG+GG	27(30.00)	273(35.27)	300	683.157±9.056	665.407–700.906	
	GG	6(6.67)	42(5.43)	58	673.104±24.066	625.936–720.2273	0.652
	AG+AA	84(93.33)	732(94.57)	816	672.929±6.204	660.769–685.089	
rs5742909	TT	3(3.33)	21(2.71)	24	685.917±24.931	637.052–734.781	0.790
	CT+CC	87(96.67)	753(97.29)	840	672.568±6.139	660.536–684.599	
	CC	64(71.11)	512(66.15)	576	668.226±7.696	653.142–683.310	0.336
	CT+TT	26(28.89)	262(33.85)	288	682.365±9.365	664.409–700.720	
rs231775	GG	44(48.89)	292(37.73)	336	657.438±10.755	636.358–678.517	0.039
	AG+AA	46(51.11)	482(62.27)	528	682.803±7.030	669.024–696.592	
	AA	9(10.00)	99(12.79)	108	693.407±13.183	666.569–718.245	0.432
	AG+GG	81(90.00)	675(87.21)	756	670.157±6.598	657.225–683.090	
rs3087243	GG	73(81.11)	599(77.39)	672	684.667±11.127	662.858–706.475	0.409
	AG+AA	17(18.89)	175(22.61)	192	669.588±7.037	655.795–683.381	
	AA	3(3.33)	21(2.71)	24	685.917±24.931	637.052–734.781	0.790
	AG+GG	87(96.67)	753(97.29)	840	672.568±6.139	660.536–684.599	

DILI: drug induced liver injury, CI: confidence intervals.

* log-rank test.

To further examine the associations of DILI with these variables, univariate and multivariate analyses were carried out with the variables age, gender, primary disease, immunosuppressive regimen, blood transfusion, HLA mismatch and rs231775 genotype (Table 6).

**Table 6:** Association between DILI and several risk factors.

Variables	Multivariable analysis		
	DILI (n = 90) (%)	Non-DILI (n = 774) (%)	*p* value
Mean age±SD	40.967±10.361	39.986±10.008	0.381
Sex			
Male/Female	56/34	480/294	0.970
Primary diseases			0.444
Chronic glomerulonephritis	87(96.67)	729(94.19)	
Polycystic kidney	2(2.22)	23(2.97)	
Pyelonephophritis	1(1.11)	22(2.84)	
Number of HLA -mismatch	2.51±0.927	2.48±0.829	0.724
Real transplantation			
lived/cadaver	7/83	93/681	0.234
Immunosuppressive regiment			0.329
CsA+MMF+Pred	55(61.11)	513(66.28)	
TAC+MMF+Pred	35(38.89)	261(33.72)	
Blood transfusion	11	93	0.955
Rejection			0.053
AR/non-AR	20/70	112/662	
rs231775 SNPs			0.040
GG	44(48.89)	292(37.73)	
AG+AA	46(51.11)	482(62.27)	

CsA: cyclosporine, MMF: mycophenolate mofetil, Pred: prednisone, TAC: tacolimous, AR: acute rejection, non-AR: non-acute rejection, DILI: drug induced liver injury.

Multivariate analyses revealed that age, gender, primary disease, immunosuppressive regimen, blood transfusion, HLA mismatch and renal transplantation were independent of DILI; however, the analyses showed that a risk factor, recipient rs231775 genotype (*p* = 0.040) was associated with DILI.

### The association of *CTLA4* haplotype and DILI

No differences in the frequencies of seven haplotypes covering the 5 SNPs existed between the DILI and non-DILI recipients (Table 7). Five haplotypes were estimated for 4 of the SNPs (excluding rs733618); the frequency of haplotype ACGG was significantly higher in the DILI group (68.9%) than in the non-DILI group (61.1%) (*p* = 0.041). No statistically significant differences were found between the DILI and non-DILI groups for the rest of the haplotypes (*p*>0.05) (Table 7).

**Table 7:** The distribution of haplotypes in 5 locus of CTLA-4 between DILI and non- DILI.

Haplotype	Frequency (%)		*χ^2^*	*p* value
	DILI	Non-DILI		
5 locus				
TACGG	65.0	59.1	2.270	0.132
CACAG	11.1	16.5	3.481	0.062
CGTAA	10.6	12.5	0.592	0.442
CGTAG	5.5	4.8	0.168	0.682
CACGG	3.9	1.9	3.103	0.078
CGCAG	1.7	2.1	0.167	0.683
TACAG	1.7	1.5	0.059	0.808
4 locus*				
ACGG	68.9	61.1	4.181	**0.041**
ACAG	12.8	17.9	2.966	0.085
GTAA	10.6	12.5	0.592	0.442
GTAG	5.5	4.8	0.168	0.682
GCAG	1.7	2.1	0.167	0.683

DILI: Drug induced liver injury,

* exclude the locus in rs 733618.

## Discussion

In kidney transplant recipients, immunosuppressive therapy is usually administered as a triple regimen and typically includes cyclosporine A (CsA)/tacrolimus (TAC)+mycophenolate mofetil (MMF)+prednisone (Pred). The mechanism of DILI has not been completely elucidated, although drugs such as CsA and steroids may induce liver cholestasis and/or hepatocyte lesions [2], causing a direct toxic effect and immune-mediated damage that may contribute to the pathogenesis of DILI. Our study revealed that the frequency of recipients carrying the rs231775 GG genotype in the DILI cohort was higher than that in the non-DILI group (*p* = 0.040). These results were consistent with the previously reported finding that the rs231775 G allele could mitigate the negative effect of *CTLA4* on T cell-mediated immune responses [23]. However, the statistical significance between groups did not hold after correction for multiple testing. This may simply be due to the sample size and, hence, lack of power to detect an association. The frequency of haplotype ACGG, including the rs231775G allele, was significantly higher in the DILI group (68.9%) than in the non-DILI group (61.1%) (*p* = 0.0409).

The fact that *CTLA4* SNPs influenced DILI may not imply that this gene product had a direct toxic effect in liver damage. From our clinical experience, while elevation of serum creatinine and AR may be diagnosed by allograft biopsy, high-dose steroids (for instance, three-day therapy with 240–500 mg/d of intravenous methylprednisolone) or increasing doses of CNI were usually administered to these patients and, to some extent, precipitated the development of DILI. The χ^2^ test showed no correlation between DILI and acute rejection (AR) (*p* = 0.053) (Table 2). Percentage of AR recipient with DILI(20/112, 17.86%) was higher than no-AR (70/662, 10.57%), but in subanalysis, no statistical differences in genotype distribution and allelic frequencies of the *CTLA4* polymorphisms were found yet in AR group and no-AR group. In our previous study [24], correlation between *CTLA4*SNPs and AR was found, whether AR is a risk of DILI or not need further discover. A larger sample size is necessary to confirm or reject the significance of this correlation. *CTLA4* SNPs influencing to DILI was possibly an internal factor. The association between *CTLA4* SNPs and liver damage has been studied in two papers. Kanno et al [17] identified that the *CTLA4* +49 (rs231775) genotype was positively associated with liver damage in primary biliary cirrhosis (PBC) in Japanese populations and that PBC patients with the G/G genotype had significantly higher serum levels of ALT, GGT, and IgM than did patients with the A/A or A/G genotype. Valenti et al [18] observed a significantly higher prevalence of the susceptible *CTLA4* allele (both in the heterozygous [OR 2.5] and in the homozygous [OR 4.6] state) in patients with alcoholic liver disease (ALD) compared to healthy subjects; this relationship was independent of age, sex and geographical origin. The *CTLA4* polymorphic G allele may confer susceptibility to ALD and, in the homozygous state, to alcoholic cirrhosis by interfering with the immune response. Ethnicity may influence a person's susceptibility to DILI [25]. For instance, African-Americans were predisposed to anticonvulsant-induced DILI, while Caucasians are particularly prone to abacavir- and flucloxacillin-induced DILI [25]. The frequency of the G allele at the *CTLA4* +49 (rs231775) locus was much higher in the Chinese population than in other populations [26]. This may indicate an even more significant role of this genetic bias. In addition, using log-rank analysis, we discovered that the rs231775 GG genotype was associated with an early onset of DILI (*p* = 0.039) (Table 5 and Figure 2).

Susceptibility factors such as age and gender were thought to confer an increased risk for the development of DILI [25]. We have discovered that age and gender are not, in fact, susceptibility factors for the development of DILI using multivariate analysis (*p* = 0.381 and 0.970, respectively), which contradicts some previous studies. In general, increased age was a risk factor for DILI (for example, an age >49 increases the risk of isoniazid hepatotoxicity) [27]. Excessive use of sodium valproate and erythromycin resulted in hepatotoxicity predominantly in children [28]. Women are widely viewed as being more likely to develop DILI, and the ALFSG has reported a female preponderance in ALF due to both paracetamol and idiosyncratic drug reactions (74% and 67%, respectively) [29]. However, a recent examination of a Spanish registry showed no overall gender difference. Rather, men, who were the predominant gender over age 60, were more likely to have a cholestatic injury, whereas women, who were the predominant gender under age 60, were more susceptible to a hepatitis-like injury [30].

Recent data suggested that DILI was associated with some immune responses, which may be influenced by human leukocyte antigen (HLA) polymorphisms. Polymorphisms of HLA-B*5701 were associated with flucloxacillin- and abacavir-induced DILI [31] and mutations in mitochondrial polymerase 1 with reactions to sodium valproate [32]. In our study, multivariate analysis demonstrated that the number of HLA mismatches was not independently associated with DILI (*p* = 0.724).

Multivariate analysis showed that a risk factor, recipient rs231775 genotype (*p* = 0.040), was associated with DILI.

In conclusion, the *CTLA4* haplotype ACGG was partially associated with the development of DILI in Chinese kidney transplant recipients. The rs231775 GG genotype may be a risk factor for immunosuppressive drug-induced liver damage in kidney transplantation. The association in the paper was statistically weak, with *p* value close to the threshold. It is possible that different populations and other statistic bias. It is need to further study on different and larger patient cohorts. The mechanism of immunosuppressant-induced DILI in renal transplantation has not been completely elucidated and needs to be studied in depth.

## Supporting Information

Table S1 **The genotype distribution of the** ***CTLA4*** **polymorphisms in AR patients with DILI and non-DILI.**
(DOC)

Table S2 **The genotype distribution of the** ***CTLA4*** **polymorphisms in non-AR patients with DILI and non-DILI.**
(DOC)

Table S3 **The allele distribution of** ***CTLA4*** **polymorphisms in AR patients with DILI and non-DILI.**
(DOC)

Table S4 **The allele distribution of** ***CTLA4*** **polymorphisms in no-AR patients with DILI and non-DILI.**
(DOC)
