# Supplementary material for: Polymorphisms in CTLA4 Influence Incidence of Drug-Induced Liver Injury after Renal Transplantation in Chinese Recipients
Source: PLoS One. 2012 Dec 21;7(12):e51723. doi: 10.1371/journal.pone.0051723 (PMC3534201; doi:10.1371/journal.pone.0051723)
Supplement: Table S1 — The genotype distribution of the CTLA4 polymorphisms in AR patients with DILI and non-DILI. (DOC) [file pone.0051723.s001.doc]

**Table S1**

The genotype distribution of the *CTLA4* polymorphisms in AR patients with DILI and non-DILI

| Locus | Genotype | Patients with DILI (n=20) n(%) | Patients with non-DILI (n=112) n(%) | Model | OR (95% CI) | *p* value |
| --- | --- | --- | --- | --- | --- | --- |
| rs733618 | TT | 14(70.00) | 70(62.50) | Dominant | 1.4(0.5~3.922) | 0.619 |
|  | CT | 5(25.00) | 32(28.57) | Recessive | 0.537(0.065~4.442) | 1.000 |
|  | CC | 1(5.00) | 10(8.92) | Codominant | 0.781(0.259~2.355) | 0.790 |
|  |  |  |  |  | 0.500(0.059~4.255) | 1.000 |
| rs4553808 | AA | 19(95.00) | 82(73.21) | Dominant | 6.951(0.891~54.208) | 0.043 |
|  | AG | 1(50.0) | 23(20.54) | Recessive | 0.84(0.778~0.907) | 0.594 |
|  | GG | 0(0.00) | 7(6.25) | Codominant | 0.859(0.351~2.105) | 0.740 |
|  |  |  |  |  | 1.232(1.121~1.353) | 0.349 |
| rs5742909 | CC | 19(95.00) | 82(73.21) | Dominant | 6.951(0.891~54.208) | 0.043 |
|  | CT | 1(50.0) | 23(20.54) | Recessive | 0.840(0.778~0.907) | 0.594 |
|  | TT | 0(0.00) | 7(6.25) | Codominant | 1.043(0.960~1.134) | 1.000 |
|  |  |  |  |  | (1.232)1.121~1.353 | 0.349 |
| rs231775 | GG | 13(65.00) | 67(59.82) | Dominant | 1.247(0.462~3.369) | 0.662 |
|  | AG | 7(35.00) | 38(33.93) | Recessive | 0.840(0.778~0.907) | 0.594 |
|  | AA | 0(0.00) | 7(6.25) | Codominant | 1.184(1.405~1.342) | 0.574 |
|  |  |  |  |  | 1.194(1.084~1.351) | 0.588 |
| rs3087243 | GG | 19(95.00) | 90(80.36) | Dominant | 4.644(0.599~36.594) | 0.197 |
|  | AG | 1(50.0) | 15(13.40) | Recessive | 0.840(0.778~0.907) | 0.594 |
|  | AA | 0(0.00) | 7(6.25) | Codominant | 1.067(0.940~1.211) | 1.000 |
|  |  |  |  |  | 1.211(1.111~1.320) | 0.597 |

DILI: drug induced liver injury, OR: odds ratio, CI: confidence intervals
