# Supplementary material for: Polymorphisms in CTLA4 Influence Incidence of Drug-Induced Liver Injury after Renal Transplantation in Chinese Recipients
Source: PLoS One. 2012 Dec 21;7(12):e51723. doi: 10.1371/journal.pone.0051723 (PMC3534201; doi:10.1371/journal.pone.0051723)
Supplement: Table S2 — The genotype distribution of the CTLA4 polymorphisms in non-AR patients with DILI and non-DILI. (DOC) [file pone.0051723.s002.doc]

**Table S2**

The genotype distribution of the *CTLA4* polymorphisms in non-AR patients with DILI and non-DILI

| Locus | Genotype | Patients with DILI (n=70) n(%) | Patients with non-DILI (n=662) n(%) | Model | OR (95% CI) | *p* value |
| --- | --- | --- | --- | --- | --- | --- |
| rs733618 | TT | 28(40.00) | 212(32.02) | Dominant | 1.415(0.854~2.345) | 0.176 |
|  | CT | 31(44.28) | 352(53.17) | Recessive | 1.073(0.544~2.115) | 0.839 |
|  | CC | 11(15.71) | 98(14.80) | Codominant | 0.667(0.389~1.143) | 0.138 |
|  |  |  |  |  | 0.850(0.407~1.777) | 0.665 |
| rs4553808 | AA | 44(62.86) | 419(63.29) | Dominant | 0.981(0.589~1.634) | 0.943 |
|  | AG | 20(28.57) | 209(31.57) | Recessive | 1.679(0.680~4.145) | 0.256 |
|  | GG | 6(8.57) | 34(5.14) | Codominant | 0.911(0.524~1.586) | 0.742 |
|  |  |  |  |  | 0.595(0.237~1.496) | 0.265 |
| rs5742909 | CC | 45(64.29) | 430(64.95) | Dominant | 0.971(0.581~1.624) | 0.911 |
|  | CT | 22(31.43) | 218(32.93) | Recessive | 2.072(0.581~7.395) | 0.217 |
|  | TT | 3(4.29) | 14(2.11) | Codominant | 0.964(0.565-1.647) | 0.894 |
|  |  |  |  |  | 0.488(0.135~1.764) | 0.225 |
| rs231775 | GG | 31(44.29) | 225(33.99) | Dominant | 1.544(0.938~2.541) | 0.086 |
|  | AG | 30(42.86) | 345(52.11) | Recessive | 0.914(0.439~1.904) | 0.810 |
|  | AA | 9(12.86) | 92(13.90) | Codominant | 0.631(0.372~1.072) | 0.086 |
|  |  |  |  |  | 1.408(0.645~3.075) | 0.388 |
| rs3087243 | GG | 54(77.14) | 509(76.89) | Dominant | 1.014(0.564~1.824) | 0.962 |
|  | AG | 14(20.00) | 139(21.00) | Recessive | 2.072(0.581~7.395) | 0.217 |
|  | AA | 2(2.86) | 14(2.11) | Codominant | 0.949(0.512~1.761) | 0.869 |
|  |  |  |  |  | 0.743(0.164~3.355) | 0.661 |

DILI: drug induced liver injury, OR: odds ratio, CI: confidence intervals
