# Supplementary material for: Polymorphisms in CTLA4 Influence Incidence of Drug-Induced Liver Injury after Renal Transplantation in Chinese Recipients
Source: PLoS One. 2012 Dec 21;7(12):e51723. doi: 10.1371/journal.pone.0051723 (PMC3534201; doi:10.1371/journal.pone.0051723)
Supplement: Table S3 — The allele distribution of CTLA4 polymorphisms in AR patients with DILI and non-DILI. (DOC) [file pone.0051723.s003.doc]

**Table S3**

The allele distribution of *CTLA4* polymorphisms in AR patients with DILI and non-DILI

| Locus | Allele | Patients with DILI (n=40) n(%) | patients with non-DILI (n=224) n(%) | OR (95% CI) | *p* value |
| --- | --- | --- | --- | --- | --- |
| rs733618 | T | 33(82.5) | 172(76.79) | 0.702(0.293~1.679) | 0.424 |
|  | C | 7(1.75) | 52(23.21) |  |  |
| rs4553808 | A | 39(97.50) | 187(83.48) | 7.717(1.028~57.939) | 0.015* |
|  | G | 1(2.50) | 37(16.52) |  |  |
| rs5742909 | C | 39(97.50) | 187(83.48) | 7.717(1.028~57.939) | 0.015* |
|  | T | 1(2.50) | 37(16.52) |  |  |
| rs231775 | G | 33(82.50) | 172(76.79) | 1.425(0.596~3.411) | 0.424 |
|  | A | 7(1.75) | 52(23.21) |  |  |
| rs3087243 | G | 39(97.50) | 195(87.05) | 5.800(0.767~43.849) | 0.059 |
|  | A | 1(2.50) | 29(12.95) |  |  |

DILI: drug induced liver injury, OR: odds ratio, CI: confidence intervals

* Bonferroni-adjusted *p* =0.075
