# Supplementary material for: Polymorphisms in CTLA4 Influence Incidence of Drug-Induced Liver Injury after Renal Transplantation in Chinese Recipients
Source: PLoS One. 2012 Dec 21;7(12):e51723. doi: 10.1371/journal.pone.0051723 (PMC3534201; doi:10.1371/journal.pone.0051723)
Supplement: Table S4 — The allele distribution of CTLA4 polymorphisms in no-AR patients with DILI and non-DILI. (DOC) [file pone.0051723.s004.doc]

**Table S4**

The allele distribution of *CTLA4* polymorphisms in no-AR patients with DILI and non-DILI

| Locus | Allele | Patients with DILI (n=140) n(%) | patients with  non-DILI  (n=1324)  n(%) | OR (95% CI) | *p* value |
| --- | --- | --- | --- | --- | --- |
| rs733618 | T | 87(62.14) | 776(58.61) | 0.863(0.603~1.235) | 0.419 |
|  | C | 53(37.86) | 548(41.39) |  |  |
| rs4553808 | A | 108(77.14) | 1045(78.93) | 0.901(0.594~1.366) | 0.623 |
|  | G | 32(22.86) | 279(21.07) |  |  |
| rs5742909 | T | 28(20.00) | 246(18.58) | 0.913(0.590~1.413) | 0.682 |
|  | C | 112(80.00) | 1078(81.42) |  |  |
| rs231775 | G | 92(65.71) | 795(60.05) | 1.275(0.885~1.839) | 0.192 |
|  | A | 48(34.29) | 529(39.95) |  |  |
| rs3087243 | G | 121(86.43) | 1157(87.39) | 0.919(0.552~1.531) | 0.746 |
|  | A | 19(13.57) | 167(12.61) |  |  |

DILI: drug induced liver injury, OR: odds ratio, CI: confidence intervals
